# Study on Rheological Behavior and Surface Properties of Epoxy Resin Chemical Grouting Material Considering Time Variation

**DOI:** 10.3390/ma12203277

**Published:** 2019-10-09

**Authors:** Zhenhua Su, Zaiqin Wang, Da Zhang, Tao Wei

**Affiliations:** 1 College of Water Conservancy and Hydroelectric Engineering, Hohai University, Nanjing 210098, China; suzhenhua@hhu.edu.cn; 2 Changjiang River Scientific Research Institute, Wuhan 430010, China; wangzq@mail.crsri.cn (Z.W.); zhangda@mail.crsri.cn (D.Z.); 3 Research Center on National Dam Safety Engineering Technology, Wuhan 430010, China

**Keywords:** rheological properties, affinity, mathematical model, infiltration and penetration ability, time variation

## Abstract

Epoxy resins are widely used for repairing cracks in stone, mortar and masonry. A main factor that influences the grouting quality is the permeability of grout. However, the permeability will deteriorate over time because of the reaction of chemical grouting materials, which will greatly affect the results of grouting. To the best of our knowledge, there are few reports that focus on the time-varying properties of viscosity and affinity of epoxy resins grouting material. In this paper, we investigate the changing rules of viscosity and affinity with time by studying the viscosity, surface tension and contact angle of the epoxy grouting material. We establish the mathematical model for the time-dependent properties of CW epoxy resin on viscosity and affinity with experimental verification. Moreover, we make a detailed discussion on the modeling of viscosity variation considering both time and temperature. The results show important guiding significance and application value for judging the grout irrigability in the construction process.

## 1. Introduction

Epoxy resin grouting material has been widely used in foundation treatment and concrete crack treatment because of its good grouting ability and high mechanical strength. One of the reasons why epoxy resins often are the materials of choice for repairing cracks in stone, mortar, and masonry is that, although they are viscous systems, they penetrate effectively into porous substrates in a way that provides an embedded network into the material [1]. The main factors that influence the grouting quality include durability, permeability, physical and mechanical compatibility. Excellent epoxy grouting materials must have high permeability, good durability and good physical and mechanical compatibility with porous media (such as old mortar and stone masonry). This paper is to study the factors affecting the permeability of epoxy grouting materials. The permeability of epoxy resin grouting material decreases with time. When the permeability is poor, only the surface layer with high mechanical strength is formed in the porous medium rather than uniform consolidation across the entire section of the element. When the permeability is relatively good, the slurry gradually penetrates into the porous medium to form a uniform consolidation unit. During the 1970s, several buildings in the United States and Europe were treated with epoxies without an adequate understanding of the viscosity-penetration problem, and the ensuing crust formation and severe discoloration gave epoxy resins a bad reputation as stone consolidants [2]. Since the 1980s, China has successfully treated muddy fractured rock masses of the Three Gorges, Xiangjiaba and Jinping hydropower stations with low viscosity and high permeability epoxy resin chemical grouting materials. The epoxy resin grouting material infiltrates and penetrates into muddy fractured rock mass to form consolidation body [3]. It can be seen that the permeability of epoxy resin grouting material directly affects the treatment effect of porous substrates.

The effects of viscosity, surface tension and contact angle of the grouting material slurry on permeability has been the subject of discussion among grouting academia and grouting workers. It is generally believed that there is a negative correlation between the viscosity and the permeability. Viscosity is one of the most important parameters affecting the rheological properties of the slurry [4]. The viscosity is normally used to characterize the rheological properties of the slurry. The rheology of the slurry of chemical grouting material reflects its fluidity under the action of external force. The better its fluidity is, the smaller the pressure loss of the slurry in the flow process and the farther its diffusion is. On the contrary, the large pressure loss of slurry in the flow process is against slurry diffusion [5,6]. The viscosity of time-varying fluids changes with time. The viscosities of chemical grouting materials such as epoxy resin grout, sodium silicate solution and chromium lignin grout all increase with time. In the process of slurry gelation, the changes of viscosities mostly conform to the function relationship of $\mu (t)=ke^{at}$ [7,8,9]. Where, μ(t) represents the slurry viscosity, t represents the time, *k* and *a* are the undetermined coefficients. The rheological parameters μ(t) of slurry have a great influence on the diffusion radius.

In fact, besides the viscosity, the surface tension of grout and the contact angle of irrigated medium are also the main factors affecting the permeability. The permeability of grout to grouted medium depends on the wettability of grout to the surface of grouted medium [10].

Young proposed the relationship between the size of the equilibrium contact angle and the interfacial tension of three phases, namely the Young equation [11,12,13], as shown in Equation (1):

(1) $$\gamma_{SG}=\gamma_{SL}+\gamma_{LG}\cos\theta ,$$

where, $\gamma_{SG}$, $\gamma_{SL}$ and $\gamma_{LG}$ were respectively the surface tension between solid-gas, solid-liquid and gas-liquid contact surfaces, and *θ* was the characteristic contact angle of solid surface.

The Young equation was the basic equation for wetting. The magnitude of the contact angle *θ* was an important index to evaluate wettability. Generally, the smaller the contact angle was, the better the wettability was. *θ* = 90° was defined as the criterion for wetting or not. When 90° ≤ *θ* ≤ 180°, it was called the non-wetting state; when *θ <* 90°, it was called the wet state; when *θ* ≤ 0°, it was called the spread wet state, at which time a certain liquid drop could be automatically spread out on a solid surface. The contact angle θ was the quantitative index to characterize the wettability between solid and liquid [14,15].

Li and Liu deduced from Young’s equation according to the second law of thermodynamics [16]

(2) $$W_{a}=\gamma_{SG}-\gamma_{SL}+\gamma_{LG}=\gamma_{LG}(\cos\theta +1),$$

(3) $$A=\gamma_{SG}-\gamma_{SL}=\gamma_{LG}\cos\theta ,$$

(4) $$S=\gamma_{SG}-\gamma_{SL}-\gamma_{LG}=\gamma_{LG}(\cos\theta -1),$$

where, $W_{a}$ was adhesion work, which represented the maximum work done by the system to the outside when the liquid and solid adhered. *A* is adhesion tension, which represented the force that spreads against the surface tension of the liquid. S was spreading factor, and its size represents the ability of a liquid to spread across a solid surface.

The above three equations showed that the adhesion work $W_{a}$, adhesion tension *A* and spreading coefficient *S* could be calculated by measuring the surface tension $\gamma_{LG}$ and contact angle *θ* of the liquid, and the wetting phenomena could be judged accordingly.

In the Young equation, $\gamma_{LG}\cos\theta$ was called the liquid-solid affinity, and the greater the affinity of the liquid was, the stronger its penetration and penetration ability was [17]. When the slurry and the irrigated medium had a certain affinity, the grout could automatically infiltrate into the irrigated medium and the irrigated medium could automatically absorb the slurry after they came into contact [18].

Imbibition has long been studied and applied in secondary oil recovery. The application mechanism of low permeability reservoirs is mainly spontaneous imbibition, which promotes water in small fractures to be sucked into reservoir matrix for oil recovery. Spontaneous imbibition displacement is a process dominated by capillary pressure. The physical process of chemical grouting is similar to water injection and oil recovery. Chemical grouting is slurry injection for water displacement and oil recovery is water injection for oil displacement [19,20,21]. The grouted medium is generally saturated. At this time, in-situ reinforcement is the grouting and drainage process. Only by using chemical grouting material to displace the pore water of the low permeability grouted medium and producing cementing and consolidation, can the effect be achieved. When the affinity of slurry to rock and soil is greater than that of pore water to rock and soil, slurry displacement of pore water is a spontaneous process of free energy reduction [17,18].

As for specific grouting materials, the permeability of grout will deteriorate with the progress of chemical grouting material reaction. The reason is related to the continuous change of viscosity, surface tension and contact angle of grout. However, the changing rules of viscosity and affinity of slurry with time have been rarely discussed in the widely published papers. These studies will provide a theoretical basis for the permeability of the slurry and important guiding significance for grouting construction and development of grouting material.

In this paper, we used the CW epoxy resin grouting material of Changjiang Academy of Sciences as the research object (CW is the trade name code of epoxy resin grouting material produced by the Yangtze River Scientific Research Institute). By measuring the time-dependent denaturation of viscosity, surface tension and contact angle of CW epoxy resin slurry, the variation of viscosity with time of CW epoxy resin slurry at different temperatures was studied. Moreover, the variation of viscosity and affinity with time of CW epoxy resin slurry was discussed.

## 2. Characteristics of CW Epoxy Resin Grouting Material 

The CW epoxy resin grouting material produced by the Yangtze River Scientific Research Institute (Wuhan, China) is a two-component grouting material composed of a new type of epoxy resin, reactive diluent, surfactant, etc. It has the characteristics of simple preparation, good irrigability, high mechanical strength, good solidification in dry and wet conditions and water. Our indoor tests and more than 20 years of engineering practice show that CW epoxy material has excellent comprehensive performance, good physical and mechanical compatibility with the irrigated body, and the entire consolidated body formed after the treatment is very durable as well [22]. It has been successfully applied in many projects, such as the strengthening treatment of F215, F1096 and F1050 fault fracture zone of Three Gorges Project in China, the seepage prevention treatment of corrosion zones in seven and eight dam sections of Jiangya Hydropower Station in Hunan Province, the treatment of basalt interlayer staggered zones in dam foundation of Xiluodu Hydropower Station, and the crack treatment of upper and lower lock heads of Xincheng Shiplock on the Jianghan Route in Hubei Province. Numerous engineering practices have proved that CW grouting material is a good reinforcing grouting material for treating bedrock and concrete cracks and muddy interlayer.

CW epoxy resin grouting material is an epoxy resin grouting material with furfural-acetone mixed diluent, which is based on the principle that furfural and acetone can react under certain conditions. Component A is composed of epoxy resin, furfural, acetone, surfactant and coupling agent, etc. Component B is composed of phenolic amine, polyamide and accelerator, etc. The main physical and mechanical properties of CW epoxy grouting material are shown in Table 1.

## 3. Materials and Methods

### 3.1. Testing Materials and Preparation Methods

CW epoxy resin grouting material, containing two components A and B (mass ratio A:B = 5:1, A:B = 6:1), was produced by the Yangtze River Academy of Sciences. Samples of each component were placed under the standard test condition of (23 ± 2) °C for 24 h, then component B was slowly poured into component A according to the recommended ratio, stirred while pouring, and fully stirred and mixed evenly.

### 3.2. Testing Equipment and Methods

(1) The viscosity of CW epoxy resin slurry was measured by NDJ-79 rotary viscometer of Shanghai Changji Geological Instrument Co, Ltd. (Shanghai, China), at different times. The measuring rotor of the NDJ-79 rotary viscometer was the number two rotor of the second unit, and the structure of the rotor was a hollow cylindrical drum with a wire hook rotating shaft. The outer diameter and height of the drum were 12.3 and 50 mm respectively. The change of viscosity of the CW epoxy resin slurry with different proportions was tested at 15, 20, 25 and 35 °C, respectively. The experiment was stopped when the viscosity exceeded 200 mPa·s.

Five hundred g of slurry was weighed by balance and stirred evenly in beaker. The viscosity of the slurry was measured by rotating viscometer at test temperature. The rotating speed of the viscometer was controlled 20%~90% of the maximum range. The viscometer was opened until the reading of the pointer is stable. The deviation of measuring again after closing the viscometer was no more than 3% until two consecutive readings were measured. The value was the average of the last two readings. At the same time, the measurement of the initial viscosity of slurry had to be completed within 5 min after the preparation of the sample, and the rotor of the viscometer had to be cleaned after the test. Each sample was measured three times, and the arithmetic average of the three measurements was taken as the test result, expressed in mPa·s. The result was accurate to 1 mPa·s. 

(2) The surface tension of the slurry at different times was measured by the Platinum plate method using the A201 automatic surface tension meter from USA KINO Industry Co, Ltd. (Boston, MA, USA), and the test temperature was 23 °C.

During the experiment, the platinum plate was immersed in the liquid, and the balance value was detected by the sensor under the immersion state, which was converted into the surface tension value and displayed.

(3) The contact angle of the slurry on the single-head single-side frosted slide (size 76 mm × 26 mm) at different times was measured by the SL200B dynamic/static contact angle meter from USA KINO Industry Co, Ltd., and the temperature was 23 °C.

The contact angle measuring instrument had to be connected to the power supply and the smooth side of the sliding glass had to be kept upwards placed on the sample stage. Then, 80 mL slurry was prepared in the beaker according to the ratio. A micro-sampler was used to extract the slurry at a depth of about 1/2 of the total liquid in the beaker. About 3 µL ± 1 µL droplets were extruded by the micro-sampler and suspended at the end of the needle. The test bench was lifted to make the slide surface contact with the droplets at the end of the needle. The test bench was moved down to transfer the droplets to the surface of the slide. The measurement of contact angle had to be completed within 60 s after the droplet was transferred. Repeated experiments were carried out in five different positions on the smooth surface of the same slide. All tests had to be completed within 10 min after slurry mixing. 

Treatment of test results were conducted in accordance with the following provisions:

The maximum value and minimum value were removed, and the arithmetic mean value of the remaining three measured values was taken as the test result, expressed in °, the result was accurate to 0.1°. When any of the measured values differed from the arithmetic mean by more than 5%, the test had to be redone.

## 4. Results and Discussion

### 4.1. Study on Viscosity Time Denaturation of CW Epoxy Slurry at the Same Temperature

**Figure 1:**
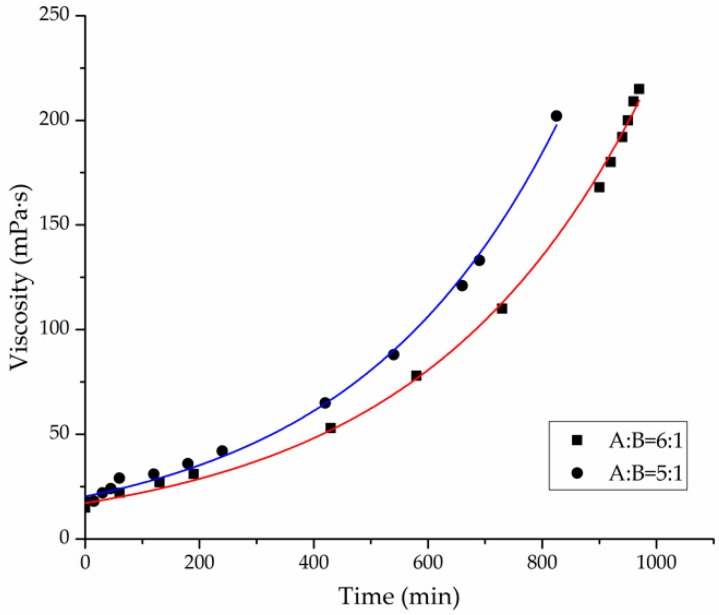
The change curve of viscosity of the slurry.

According to the test results, the viscosity of CW epoxy resin slurry with different proportions changed with time at 20 °C. The viscosity test results of CW epoxy resin with mass ratio of A:B = 5:1 and A:B = 6:1 were shown in Figure 1. It could be seen that the viscosity of CW epoxy resin slurry gradually increased with time. The initial viscosity of the slurry was about 15 mPa·s, which changed more slowly before about 450 min. When the slurry was mixed and stirred for more than 450 min, the viscosity of the slurry increased gradually. The viscosity of grout with ratio A:B = 5:1 grew faster than that of grout with ratio A:B = 6:1.

By fitting the viscosity-time relationship of the slurry, it is known that the viscosity of the CW epoxy slurry changes with time, which was in accordance with the exponential function of $\mu (t)=ke^{at}$.

According to the fitting results, for the slurry with mass ratio A:B = 5:1 (*k* = 20.278, *a* = 0.0028), the fitting correlation coefficient of *R*^2^ was 0.997. Accordingly, we could get the model of viscosity of CW epoxy resin slurry with mass ratio A:B = 5:1 as follows:

(5) $$\mu \left(t\right)=20.278e^{0.0028t}.$$

For the grout with mass ratio of A:B = 6:1, *k* = 17.154, *a* = 0.0026, the correlation coefficient of fitting was *R*^2^ = 0.998. Similarly, we could get the model of viscosity of CW epoxy resin slurry with mass ratio A:B = 6:1 as follows:

(6) $$\mu \left(t\right)=17.154e^{0.0026t}.$$

Equations (5) and (6) are statistical models. We used Origin software to fit the measured viscosity data with time. The correlation coefficient *R*^2^ of the model was more than 0.99, and the fitting correlation was good. At the same time, after a large number of laboratory tests, it was verified that the measured value was in good agreement with the calculated value of the model, and the error was in a very small range. Origin was used to check the significance of the model coefficients. The significance Prob > F value was far less than 0.05, so the significance was very good.

### 4.2. Time-Dependent Viscosity Test of CW Epoxy Slurry at Different Temperatures

#### 4.2.1. Effect of Temperature on Rheological Characteristics of Slurry

**Figure 2:**
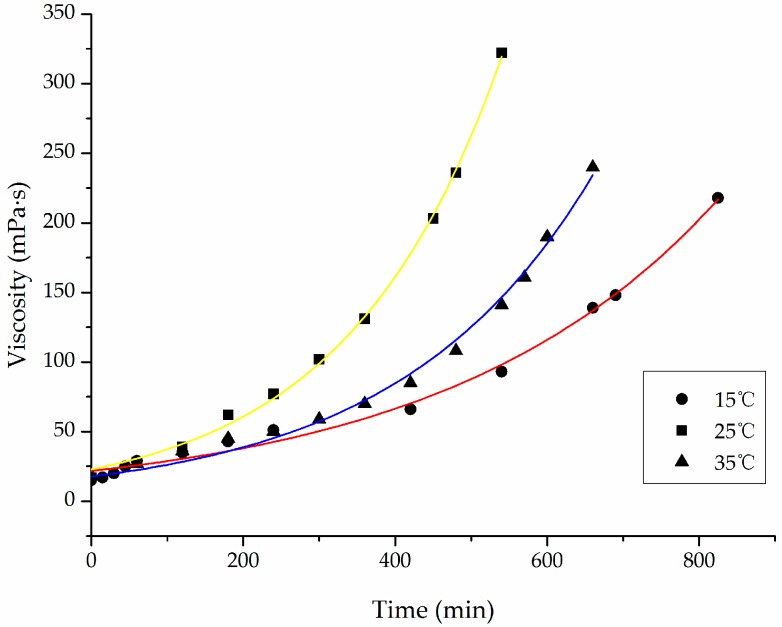
The viscosity-temperature-time curve of CW 5:1 slurry.

**Figure 3:**
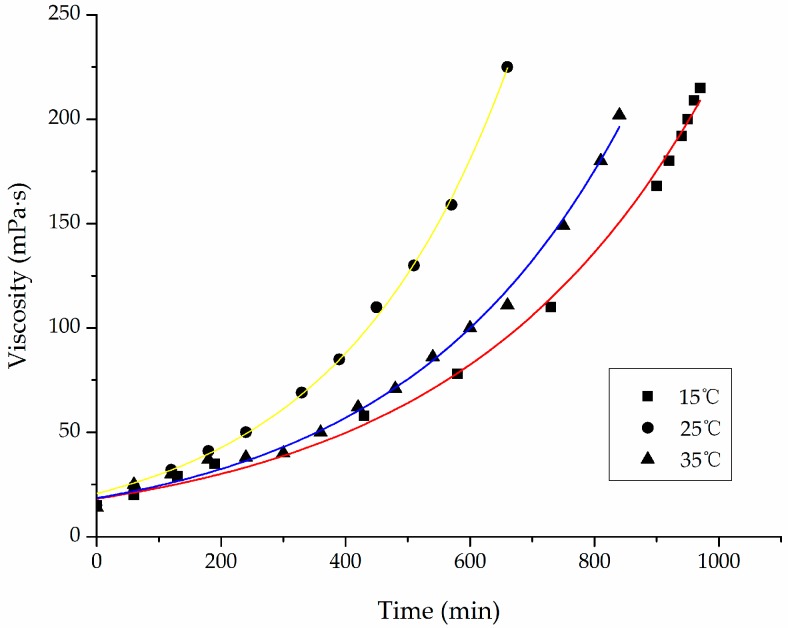
The viscosity-temperature-time curve of CW 6:1 slurry.

The most commonly used ratios of CW epoxy resin grouting material (A:B = 5:1, A:B = 6:1) were selected to study the changes of their viscosity over time at 15, 25 and 35 °C, respectively. The viscosity time-varying curves of the two slurries at different temperatures were shown in Figure 2 and Figure 3.

From Figure 1, it could be seen that the viscosity of A:B = 5:1 and A:B = 6:1 slurries varies with time at the same temperature, and increased with time, which conformed to the exponential function relationship of $\mu (t)=ke^{at}$. At the same time, the three kinds of slurries had the lowest viscosity at 15 °C. The viscosity of CW511 slurry with ratio 5:1 and A:B = 6:1 at 25 °C was higher than that at 35 °C.

Comprehensive analysis showed that the viscosity of CW slurry changed with temperature and time. Zhu et al. pointed out that there are physical and chemical factors affecting the viscosity of epoxy resin system. On the one hand, the increase temperature would intensify the movement of molecules and increase the fluidity of slurry. Therefore, the slurry viscosity would be reduced. On the other hand, the increase temperature would also accelerate the chemical reaction between the slurry components and increase the polymerization degree. As a consequence, the slurry flow would be hindered, and the viscosity would be increased [23]. It could be seen from the curves shown in Figure 2 and Figure 3 that the viscosity of the slurry at 15 °C at the same time was less than that at 25 °C. The main reason was that the increase of viscosity caused by chemical change was greater than decrease of viscosity caused by physical change. The increase of viscosity caused by polymer chemical reaction played a leading role. The viscosity of slurry at the same time at 25 °C was greater than that at 35 °C. The main reason was that the decrease of viscosity caused by physical change was greater than increase of viscosity caused by chemical change. The decrease of slurry viscosity caused by temperature rise played a leading role. 

#### 4.2.2. Discussion on the Relationship between Slurry Viscosity and Temperature-Time

Assuming that the slurry conformed to the Dual-Arrhenius viscosity model [24,25,26,27,28], the model parameters *k*, at different temperatures, were determined by fitting the temperature curves of the viscosities of Figure 2 and Figure 3 according to the formula $\mu (t)=ke^{at}$. 

The curves of Figure 2 and Figure 3 were fitted according to the formula $\mu (t)=ke^{at}$ to determine the model parameters *k*, *a* at different temperatures (see Table 2).

It could be seen from the table that the fitting correlation coefficient *R*^2^ at different temperatures was greater than 0.990, which indicated that the simulation correlation of this viscosity model was very good. Significance test had been done in Table 2 as well. Prob > F value is less than 0.05, which was very significant.

It was indicated that the chemical rheological model of the resin system conformed to the Dual-Arrhenius viscosity model.

To improve the viscosity time-varying function of the shape such as $\mu (t)=ke^{at}$, the following expression could be obtained:

(7) $$k=k_{1}\exp\left(k_{2}/T\right),$$

(8) $$a=k_{3}\exp\left(k_{4}/T\right),$$

where, $k_{1}$, $k_{2}$, $k_{3}$ and $k_{4}$ were all chemical rheological model parameters of thermosetting resins, T was the test temperature.

In order to determine the values of the rheological model parameters $k_{1}$, $k_{2}$ of the initial viscosity *k*, the Equation (7) was transformed as follows:

(9) $$\ln k=\ln k_{1}+k_{2}/T.$$

**Figure 4:**
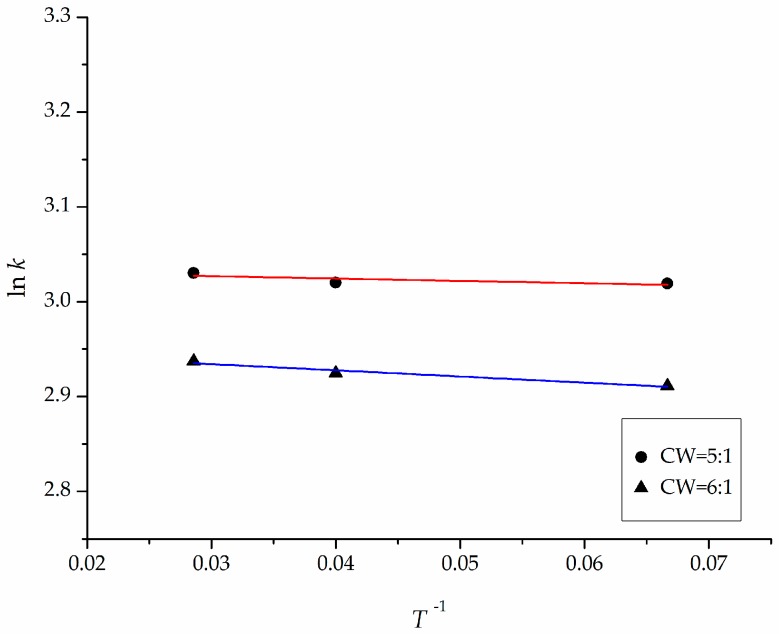
The lnk-1/T curve.

Lnk-1/T curve was made for the test data, as shown in Figure 4.

From the results of the linear analysis, the curve equations of Table 3 could be obtained, and the linear fitting correlation coefficients were all greater than 0.9, indicating that the linear relationship between lnk and 1/T was good.

From Table 3, the values of parameters $k_{1}$ and $k_{2}$ of three groups of rheological models of slurry initial viscosity could be obtained, and their corresponding initial viscosity model equations could be derived, as shown in Table 4.

In order to determine the values of rheological model parameters $k_{3}$ and $k_{4}$, Equation (8) was transformed as follows:

(10) $$\ln a=\ln k_{3}+k_{4}/T.$$

**Figure 5:**
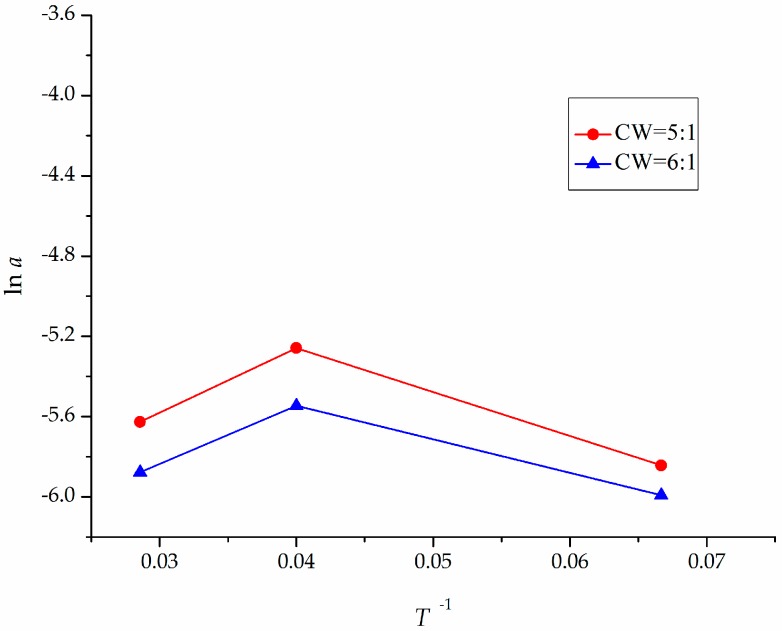
The lna-1/T curve.

Lna-1/T curve was made for the test data, as shown in Figure 5.

From Figure 5, it could be seen that the lna-1/T curves of A:B = 5:1 and A:B = 6:1 slurries did not conform to the linear relationship. This meant that the results did not conform to Equation (10).

Jianhua Qiu et al. used butyl glycidyl ether as the active diluent to dilute epoxy resin and polyamide as curing agent to prepare epoxy resin grouting material [29]. They used Dual-Arrhenius equation to establish the function of viscosity, temperature and time of epoxy resin grouting material. The reason why there is no way to establish the function of slurry viscosity-temperature-time for CW epoxy grouting is presented below.

From the analysis of reaction mechanism, the grouting materials prepared by Jianhua Qiu et al. are only the product of gradual polymerization reaction of epoxy groups and curing agents [29]. In addition to epoxy resin, CW grouting material formula also contains furfural, acetone and other components. The system includes not only the gradual polymerization of epoxy group with curing agent, but also the reaction of a-hydrogen between furfural and acetone, as well as the reaction of furfural and acetone with amine in curing agent [30].

The specific reaction is as follows:

1. Cross-linking curing reaction of epoxy resin with amine.

**Scheme 1:**
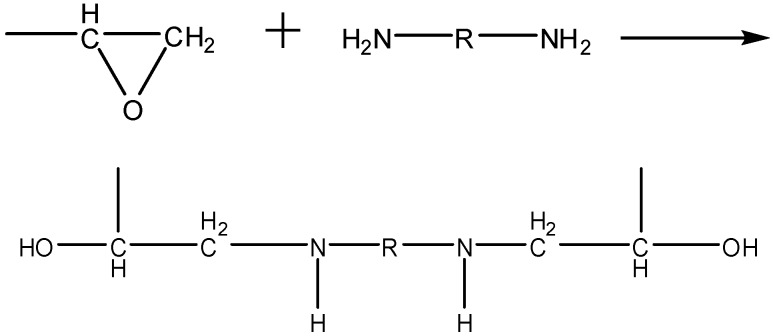
Reaction of primary amine with epoxy group.

**Scheme 2:**
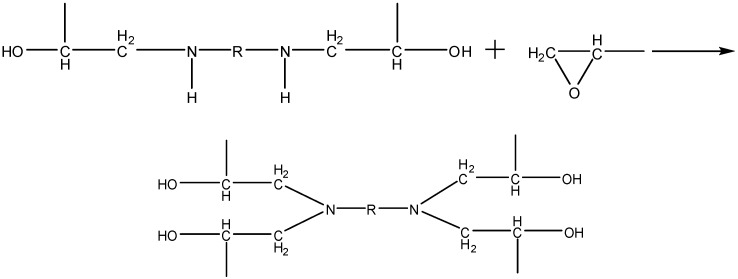
Reaction of secondary amine with epoxy group.

In the first step, the primary amine reacts with the epoxy group in the epoxy resin to bring the epoxy group to form a secondary amine. The reaction formula is as follows (Scheme 1):

In the second step, secondary amines continue to react with epoxy groups to form tertiary amines and large reticulated polymers. The reaction formula is as follows (Scheme 2):

Because of the large substituents on nitrogen atoms, the formation of tertiary amines makes it difficult to further open the epoxy ring and promote the epoxy-epoxy polymerization.

2. Condensation reaction of furfural with acetone in alkaline medium.

The reaction produces furan methylene acetone in two steps.

**Scheme 3:**
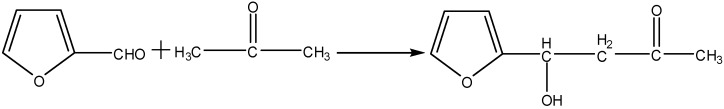
Reaction of furfural with acetone.

**Scheme 4:**
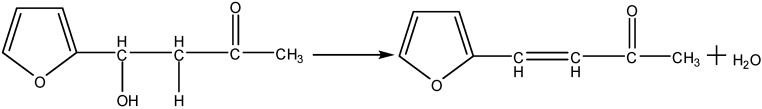
Dehydration reaction.

**Scheme 5:**
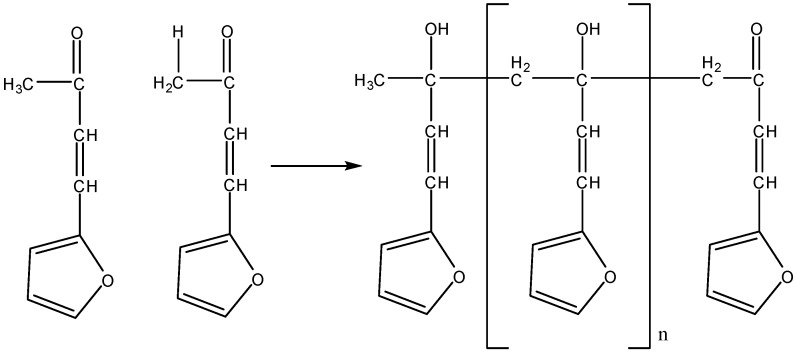
Reaction of forming furfural-acetone resin.

The first step is the addition reaction. The reaction formula is as follows (Scheme 3):

The second is the dehydration reaction. The reaction formula is as follows (Scheme 4):

When the molar ratio of furfural to acetone is less than 1 in excess of acetone, the third step of reaction polymerization will take place to produce furfural-acetone resin. The reaction formula is as follows (Scheme 5):

3. Reaction of Amine with Furfural and Acetone.

**Scheme 6:**
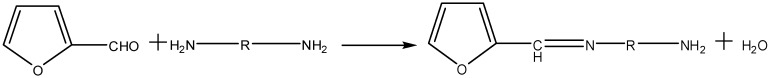
Reaction of furfural with primary amine.

**Scheme 7:**
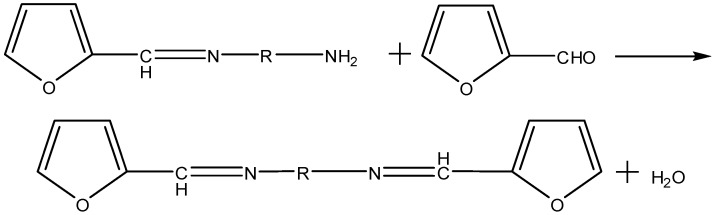
Reaction of aldehyde imine with furfural.

**Scheme 8:**
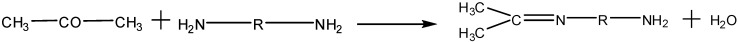
Reaction of acetone with primary amine.

**Scheme 9:**
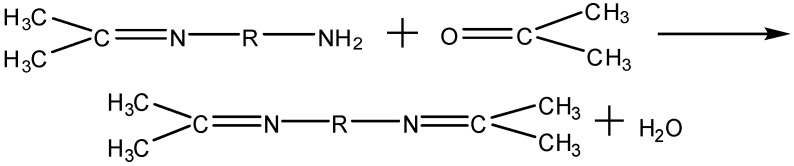
Reaction of ketimine with acetone.

Amine reacts with furfural as follows (Scheme 6):

Amine continues to react with furfural as follows (Scheme 7):

Amine reacts with acetone as follows (Scheme 8):

Amine continues to react with acetone as follows (Scheme 9):

It can be seen that the reaction of the system is complex. These exothermic reactions will produce unpredictable temperature changes. The difference of reaction process and the change of temperature may further affect the rheological properties of materials. This may be the reason why CW epoxy grouting material cannot establish the function relationship between the viscosity of slurry, temperature and time.

### 4.3. The Surface Tension of the Slurry Changes With Time

**Figure 6:**
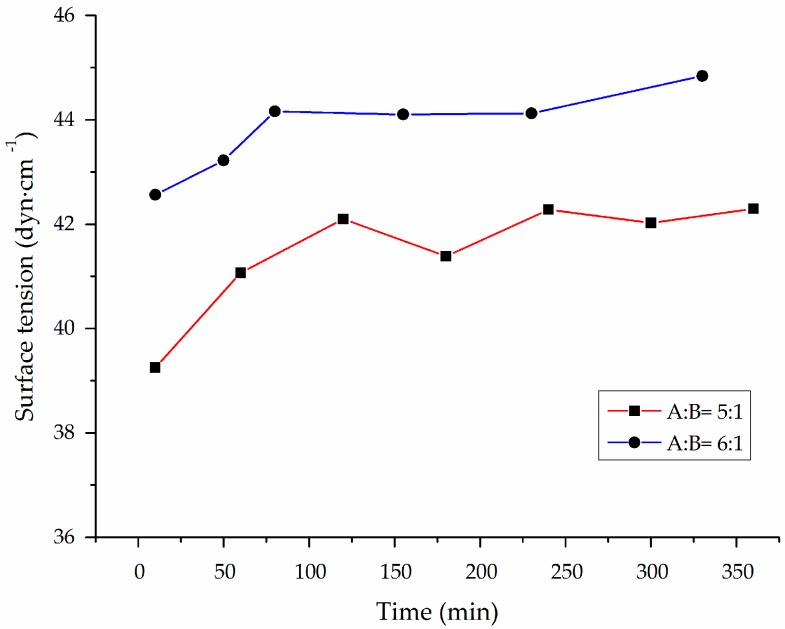
The change curve of surface tension the slurry.

The surface tension test results for the CW epoxy resin slurry with mass ratios A:B = 5:1 and 6:1 were shown in Figure 6.

As shown in Figure 6, the surface tension of the slurry with different mass ratios was different. The initial surface tension of the slurry with mass ratio 5:1 was 39 dyn·cm^−1^, while the initial surface tension of the slurry with mass ratio 6:1 was 42 dyn·cm^−1^. For slurry with a mass ratio of 5:1, the surface tension tended to increase rapidly in about 60 min. After more than 60 min, the change of surface tension tended to be stable and reached equilibrium state. The surface tension value under equilibrium state was about 42 dyn·cm^−1^. For the slurry with a mass ratio of 6:1, the surface tension increased rapidly before 75 min and reached equilibrium after 75 min. The surface tension in equilibrium state was about 44 dyn·cm^−1^.

### 4.4. The Contact Angle of the Slurry Changes With Time

**Figure 7:**
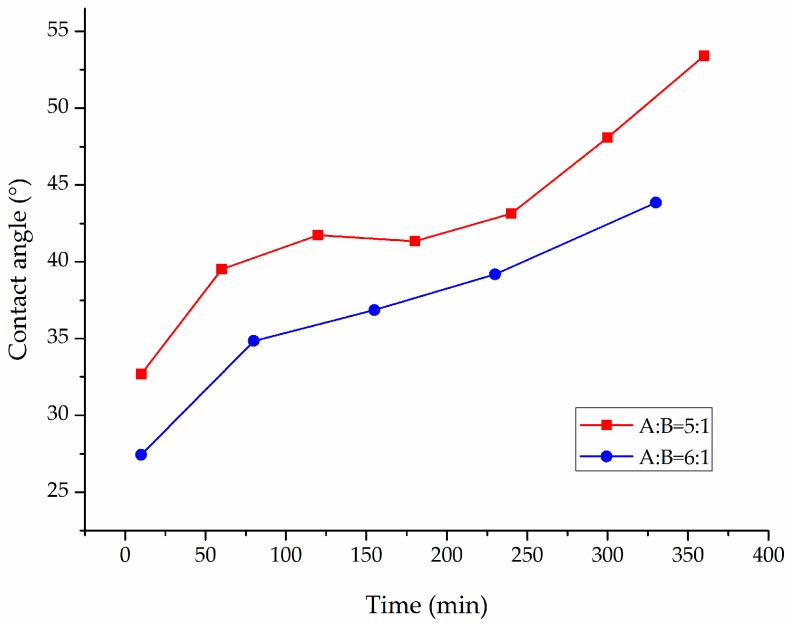
The change curve of contact angle of the slurry.

The contact angle results of the CW epoxy resin slurries with mass ratios 5:1 and 6:1 taken at different times were shown in Figure 7. 

As shown in Figure 7, the contact angle of different mass ratios of CW epoxy resin slurry samples tested at different time increased with time. The main reason for this trend was that the slurry viscosity increases continuously. The slurry with a mass ratio of A:B = 5:1 maintained a higher growth rate in the first 60 min, and the change of the contact angle gradually became slow after 60 min. The slurry with mass ratio A:B = 6:1 maintained a high growth rate in the first 75 min, and the growth gradually slowed down after 75 min. From the results of the contact angle, the slurry with mass ratio of A:B = 6:1 had a lower contact angle and a slower rate of change at an initial stage, which was more advantageous for the infiltration and penetration of the slurry.

### 4.5. Mathematical Model Establishment of the Affinity Of Slurry With Time

**Figure 8:**
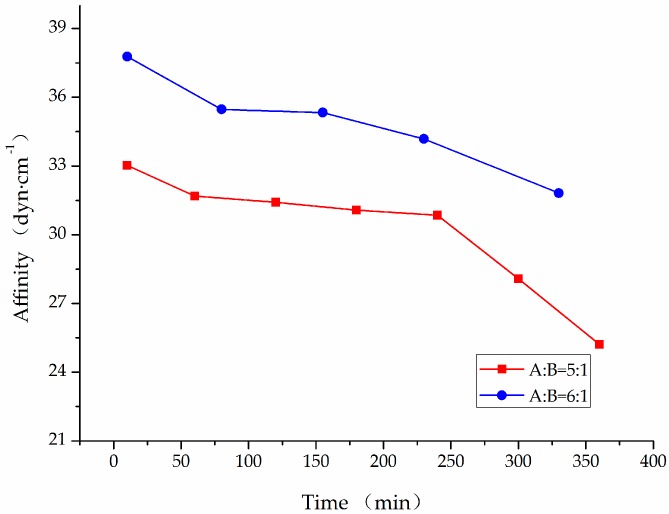
The change curve of affinity of the slurry.

$\sigma_{L}\cos\theta$ was called liquid-solid affinity, where $\sigma_{L}$ was the solid/slurry interfacial tension and *θ* was the liquid/solid phase contact angle. The greater the affinity of the liquid was, the stronger its infiltration and permeation ability was. Based on the measured surface tension and contact angle data, the curves of the two paste affinities were obtained, as shown in Figure 8.

**Figure 9:**
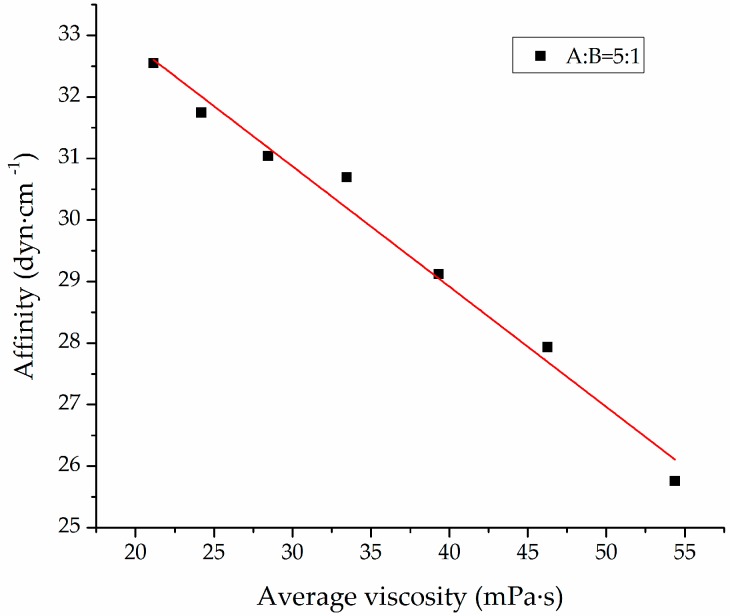
The relationship between average viscosity and affinity of slurry (A:B = 5:1).

**Figure 10:**
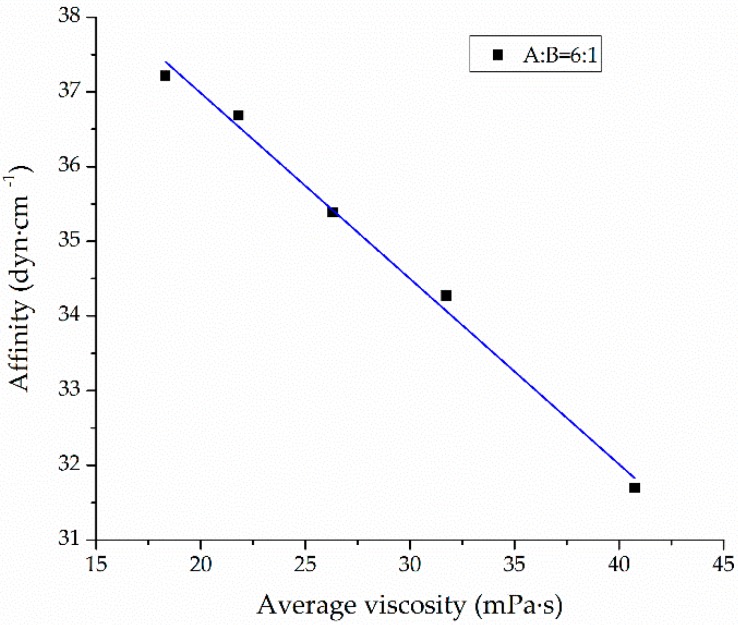
The relationship between average viscosity and affinity of slurry (A:B = 6:1).

As shown in Figure 8, the affinity of slurry decreased with time. Because affinity characterized the ability of infiltration and permeability of slurry, it indicated that the infiltration and permeability of slurry would also deteriorate with time. The viscosity of the slurry in Figure 1 increased with time, and the affinity of the slurry in Figure 8 decreased with time. The relationship between the average viscosity and affinity of two different slurries was obtained by linear fitting, as shown in Figure 9 (A:B = 5:1) and Figure 10 (A: B = 6:1).

By means of multi-point linear fitting, we obtained the viscosity-affinity relationship of the slurry with mass ratio A:B = 5:1 in Equation (5), and the correlation *R*^2^ = 0.985. The average viscosity-affinity relationship was presented below:

(11) $$T=36.74-0.2\bar{\mu },$$

where, T denoted the affinity and $\bar{\mu }$ denoted the average viscosity of the slurry.

The viscosity-affinity relationship of slurry with mass ratio A:B = 6:1 was shown in Equation (6), correlation R^2^ = 0.994. The average viscosity-affinity relationship was presented below:

(12) $$T=42-0.25\bar{\mu },$$

where, T was affinity and $\bar{\mu }$ was the average viscosity of the slurry.

By substituting Equation (5) into Equation (11) and introducing integral algorithm, the affinity-time mathematical model of slurry with mass ratio A:B = 5:1 could be obtained:

(13) $$T=-0.2\frac{\int_{0}^{t_{0}}20.278e^{0.0028t}dt}{t_{0}}+36.74.$$

By substituting Equation (6) into Equation (12) and introducing integral algorithm, the affinity-time mathematical model of slurry with mass ratio A:B = 6:1 could be obtained:

(14) $$T=-0.25\frac{\int_{0}^{t_{0}}17.154e^{0.0026t}dt}{t_{0}}+42,$$

where, $t_{0}$ represented the testing time point. The $\int_{0}^{t_{0}}ke^{at}dt/t_{0}$ represented the average viscosity from time point 0 to $t_{0}$.

**Figure 11:**
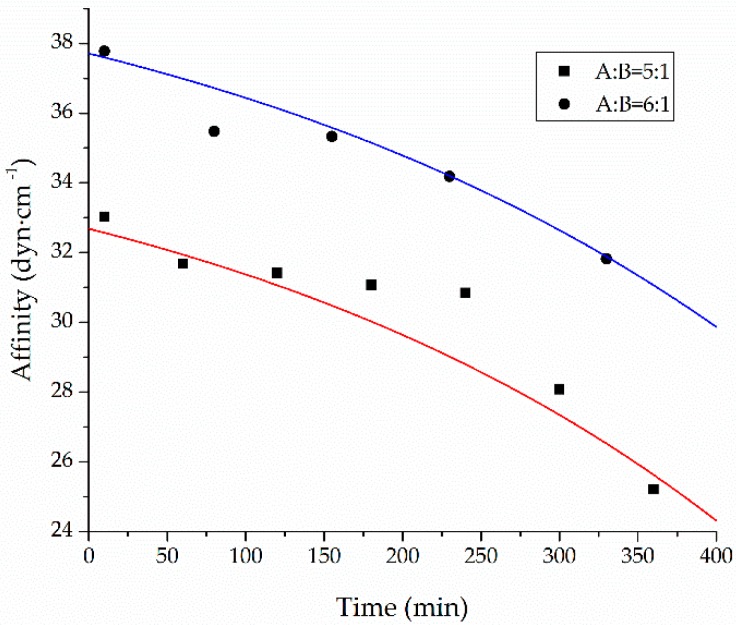
The comparison of the experimental data and the model curve of the slurry affinity time.

As shown in Figure 11, the previously measured surface tension and contact angle data were calculated to obtain the results of the two slurry affinity changes with time and the mathematical model curves of Equations (13) and (14) were compared. It could be seen from the figure that the mathematical model curve and the actual experimental data had a high degree of agreement, which also showed the correctness of the mathematical model. From the mathematical model of slurry affinity, the slurry had a higher affinity when the initial viscosity was lower. When the affinity was 0, the slurry had little infiltration and penetration ability. For the mass ratio A:B = 5:1 slurry, the reaction time was about 13 h when its affinity was 0, and for the mass ratio A:B = 6:1 slurry the reaction time was about 16 h when its affinity was 0. At the same time, it could be seen that the affinity of A:B = 6:1 slurry was better, and its infiltration and permeability were better, too.

## 5. Conclusions

This paper studied on the viscosity, surface tension and contact angle time-varying properties of CW epoxy resin grout material with mass ratio of A:B = 5:1 and 6:1. Besides, the law of grout affinity changing with time was also discussed. The mathematical model of viscosity and affinity changing with time of CW epoxy resin grout was established and the following conclusions were drawn:

1. The chemical rheological characteristics of grouting material at different temperatures were discussed by using the double Arrhenius equation. The time-varying of CW epoxy grouting material’s slurry viscosity accorded with the exponential function law. The parameter A was the initial slurry viscosity, and the parameter B represented the growth rate of viscosity of slurry. The smaller the B value, the slower the slurry viscosity growth. The models of the viscosities of CW epoxy resin slurries with the changing with time at mass ratios of A:B = 5:1 and 6:1 were presented as follows, respectively: $\mu \left(t\right)=20.278e^{0.0028t}$ and $\mu \left(t\right)=17.154e^{0.0026t}$.
2. The surface tension of slurries with different mass ratios was varied and increased with the increase of reaction time until reaching equilibrium state. The initial surface tension of the slurry with a mass ratio of 5:1 was 39 dyn·cm^−1^, while that of the slurry with a mass ratio of 6:1 is 42 dyn·cm^−1^.
3. The contact angle of CW epoxy resin slurry with different mass ratios increased with the increase of time. The contact angle of CW epoxy resin slurry with a mass ratio of A:B = 6:1 was lower than that of CW epoxy resin slurry with a mass ratio of 5:1 in the same time.
4. The affinity of CW epoxy resin slurry with different mass ratios decreased with the increase of time. Because affinity reflected the infiltration and penetration ability of slurry, the infiltration and penetration ability of slurry also decreased with the increase of time. The mathematical models for the affinity of the CW epoxy resin slurry with mass ratio of A:B = 5:1 and mass ratio of A:B = 6:1 as a function of time were respectively: $T=-0.2\frac{\int_{0}^{t_{0}}20.278e^{0.0028t}dt}{t_{0}}+36.74$ and $T=-0.25\frac{\int_{0}^{t_{0}}17.154e^{0.0026t}dt}{t_{0}}+42$. The model in this paper is mainly applicable to epoxy resin grouting materials cured at room temperature with furfural-acetone mixture diluter. The models for other resin materials and different curing conditions may be studied with reference to the method in this paper.

## Figures and Tables

**Table 1:** Physical and mechanical properties of CW epoxy grouting material.

Items		6:1	5:1
Initial setting time (h)		65	46
Slurry density (g/cm^3^)		1.06	1.07
Compressive strength (MPa)		62	68
Shear strength (MPa)		12	15
Tensile strength (MPa)		16	20
Bond strength (MPa)	Dry	4.8	5.2
	Wet	4.2	4.0

**Table 2:** The undetermined coefficient and correlation coefficient of rheological properties fitting function of slurry at different temperatures.

Temperature (°C)	Correlation Coefficient	A:B = 5:1	A:B = 6:1
15 °C	*k*	20.29	18.36
	*a*	0.0030	0.0025
	*R* ^2^	0.9933	0.9967
25 °C	*k*	20.55	18.69
	*a*	0.0049	0.0025
	*R* ^2^	0.9983	0.9981
35 °C	*k*	20.67	18.82
	*a*	0.0031	0.0027
	*R* ^2^	0.9924	0.9954

**Table 3:** The lnk-1/T curve equation and fitting coefficient.

Slurry	Curve Equation	Fitting Coefficient
A:B = 5:1	lnk=−0.4837/T+3.0423	0.9352
A:B = 6:1	lnk=−0.6518/T+2.9538	0.9576

**Table 4:** The model equation of initial viscosity.

Slurry	Initial Viscosity Model Equation
A:B = 5:1	k=20.95exp(−0.4837/T)
A:B = 6:1	k=19.18exp(−0.6518/T)
